# Supplementary material for: Parents’ Perceptions about Salt Consumption in Urban Areas of Peru: Formative Research for a Social Marketing Strategy
Source: Nutrients. 2020 Jan 8;12(1):176. doi: 10.3390/nu12010176 (PMC7019816; doi:10.3390/nu12010176)
Supplement: Supplementary file 1 [file nutrients-12-00176-s001.zip › Table S1_Parents-perceptions-salt-consumption_Formative research.docx]

**Table S1**. (Supplementary Material)

Summary results quantitative phase

|  |  |  | Men | Women | Total | *p* |
| --- | --- | --- | --- | --- | --- | --- |
| *Cooking practices* | Decides to cook at home (n=217) | Mother | 65.6% | 86.7% | 83.9% | 0.049 |
|  |  | Dad | 3.4% | 1.6% | 1.8% |  |
|  |  | Grandmother | 13.8% | 5.3% | 6.5% |  |
|  |  | Housekeeper | 0.0% | 1.1% | 0.9% |  |
|  |  | Others | 17.2% | 5.3% | 6.9% |  |
|  | Before deciding to cook, receives suggestions from… (n=221) | Couple | 56.2% | 32.8% | 36.2% | 0.075 |
|  |  | Children | 9.4% | 20.1% | 18.5% |  |
|  |  | Grandparents | 6.3% | 6.9% | 6.8% |  |
|  |  | Nobody | 28.1% | 40.2% | 38.5% |  |
|  | Most important category when deciding what to cook (n=220) | Weather | 40.0% | 15.8% | 19.1% | 0.018 |
|  |  | The taste | 6.7% | 8.9% | 8.6% |  |
|  |  | The money | 6.7% | 13.2% | 12.3% |  |
|  |  | Healthy | 46.6% | 62.1% | 60.0% |  |
| *Perception of their consumption of salt and seasonings* | You would be willing to reduce his salt intake in his food if that doesn't sacrifice the taste (n=218) | Yes | 79.4% | 65.1% | 67.0% | 0.058 |
|  |  | Do not | 0.0% | 0.5% | 0.5% |  |
|  |  | Maybe | 10.3% | 11.1% | 11.0% |  |
|  |  | I eat with little salt | 10.3% | 23.3% | 21.5% |  |
|  | To enhance the taste of your meals use (n=212) | Artificial seasonings | 50.0% | 28.6% | 31.6% | 0.033 |
|  |  | Add more salt | 0.0% | 3.3% | 2.8% |  |
|  |  | Natural condiments | 46.7% | 57.1% | 55.7% |  |
|  |  | Others | 3.3% | 11.0% | 9.9% |  |
|  | Frequency of use of artificial seasonings (n=229) | Daily / Interdiary | 30.3% | 22.4% | 23.6% | 0.085 |
|  |  | Twice a week | 24.2% | 13.3% | 14.8% |  |
|  |  | Rarely | 33.4% | 33.2% | 33.2% |  |
|  |  | I do not use | 12.1% | 31.1% | 28.4% |  |
| *Knowledge about the consequences of high salt consumption* | Diseases that can develop due to excessive salt consumption (n=231) ^a^ | Hypertension | 79.4% | 79.7% | 79.7% | - |
|  |  | Diabetes | 32.4% | 41.1% | 39.8% |  |
|  |  | Cancer | 17.6% | 26.9% | 25.5% |  |
|  |  | Heart attack | 32.4% | 41.6% | 40.3% |  |
|  |  | Don't know | 11.8% | 9.6% | 10.0% |  |
| *Motivators and barriers to reduce salt intake* | If you find out that your child can acquire a chronic disease, by consuming a lot of salt, you feel … (n=214) | Fear | 19.4% | 9.8% | 11.2% | 0.367 |
|  |  | Concern | 77.4% | 82.5% | 81.8% |  |
|  |  | Guilt | 3.2% | 5.5% | 5.1% |  |
|  |  | Others | 0.0% | 2.2% | 1.9% |  |

^a^ The participants can select more of one option. *p* = Significance value of the Chi-square test
