# Supplementary material for: Parents’ Perceptions about Salt Consumption in Urban Areas of Peru: Formative Research for a Social Marketing Strategy
Source: Nutrients. 2020 Jan 8;12(1):176. doi: 10.3390/nu12010176 (PMC7019816; doi:10.3390/nu12010176)
Supplement: Supplementary file 1 [file nutrients-12-00176-s001.zip › supplementary materials.docx]

**UNIVERSIDAD PERUANA CAYETANO HEREDIA**

**CRONICAS CENTER OF EXCELLENCE IN CHRONIC DISEASES**

**IN-DEPTH INTERVIEW QUESTIONS**

**Study Title:** Scaling-up and evaluating salt reduction policies and programs in Latin American countries.

**Principal Investigators:** J. Jaime Miranda y Lorena Saavedra

**SIDISI No.:** 66215

**PI Version Date:** November 03, 2017 (Version 2.0)

*Please, introduce yourself orally before starting:*

*“Good morning/afternoon, my name is (say your name and show your badge). I am part of the Universidad Peruana Cayetano Heredia’s staff. We are carrying out some interviews to gather your perceptions and knowledge regarding salt intake, opinions and practices about the use of salt, as well as your predisposition to reduce salt in food, and to collect ideas and suggestions about how people might change their habits of salt consumption. If you decide to participate, we will request part of your time to be interviewed by our personnel. This interview will take approximately one hour and will be held in a private location.*

*Before inviting you, I would like to ask you some questions about your daily life and then start the interview.”*

**1. Let’s talk about food and salt in your family...**

- What is your children’s favorite dish?
- Are you the one who decide what to cook at home?
  - (If the answer is negative) who decide?
- You mentioned that............ is your children’s favorite dish.
  - Imagine that you are going to prepare it, tell me the steps you follow once you bought the ingredients.
  - (If it doesn’t have salt) and the salt? In what moment or moments do you add it?
  - How do you add it? What do you use? (If there is no answer) Do you use spoon or ladle, a little of salt or salt shaker?
  - Usually, what do you do to realize that you are adding the correct amount of salt that your family like?
- In your house, when the food is served, do your family used to add more salt?
  - Do they try the prepared dish before adding the salt?
- Usually, what do you use to give flavor to your food?
  - (If they don’t answer seasonings) Do you use seasonings?
  - From that list, what kind of seasonings do you use?
  - Do you know which are the natural condiments that add flavor to the food? Can you mention some of them?
    - Which one do you use and how frequently do you use them?

**2. Let’s talk about decisions we make when cooking...**

- What do you take into account when deciding what to cook: the time, the money, the flavor, healthy food?
  - Can you order them from the top to the bottom?
- Usually what contains the.......... The ingredient that is always present is.............
  - House breakfast?
  - House lunch?
  - House dinner?
- How frequently do the members of your family eat outside your house?
  - Do they take the home-made food to their jobs? (Recipe or canner)
  - When they eat outside? At breakfast, lunch or dinner?
  - Why do they eat outside? Give me some reasons.

**3. Let’s talk about salt and health...**

- What did you hear about the relationship between salt consumption and health?
  - Do you know how many diseases can be developed for the excessive consumption of salt?
  - What did you hear about arterial hypertension and the relationship between the consumption of salt?
  - If I tell you that your kids can develop diseases such as hypertension, diabetes or cancer for the consumption of salt, how do you feel?
- Do you know what is the good thing about reducing the consumption of salt?
  - Can you give me some benefits?
- Have you ever tried to reduce the consumption of salt in your house?
  - No, why not?
    - Imagine that you want to do it, what would you do?
    - How many of them would like the change?
    - How many of them wouldn’t like to change?
    - How many of them support your decision?
  - Yes, why did you try to reduce the consumption of salt?
    - What did you do to reduce the consumption of salt in your house?
    - How do the members of your family react to the reduction of salt?
    - About the actions you have made, which ones were easier to do?
    - Which ones where harder to do?
- Imagine that there is a lack of salt in the market and since now we must to cook with less salt. What would you do to cook with a little bit of salt and maintain the flavor of the food?
  - (If there are few answers) what ingredients would you use to replace the salt?

**4. Let’s talk about some free ideas to know about salt and health...**

- What do you think about the following ideas?
  - We propose you to use a salt-restriction spoon to control the amount of salt that you use in the preparation of your foods.
    - Do you like the idea?
    - What do you dislike about the idea?
    - Would you use it?
  - We propose to use a recipe book that inform you how to cook with less salt and use natural condiments without sacrifice the flavor in your foods.
    - Do you like the idea?
    - What do you dislike about the idea?
  - We propose to participate in virtual cooking classes or in cooking sessions to learn how to cook with little amount of salt, reducing cooking time and without sacrifice the flavor of your foods.
    - Do you like the idea?
    - What do you dislike about the idea?
    - Would you attend or to see it?
  - We propose one chart/one magnet for the refrigerator that helps you to classify the food that you add in low, medium and high amounts of salt.
    - Do you like the idea?
    - What do you dislike about the idea?
    - Would you use it?

**5. Let´s talk about the job that you realize in the municipality and the school of your kids.**

- Do you know if the Municipality has done something to reduce the consumption of salt in the population?
- Have the school ever had some actions to reduce the consumption of salt?
  - What does the school could do?

**UNIVERSIDAD PERUANA CAYETANO HEREDIA**

**CRONICAS CENTER OF EXCELLENCE IN CHRONIC DISEASES**

**GUIDE FOR FOCUS GROUP**

**Study Title:** Scaling-up and evaluating salt reduction policies and programs in Latin American countries.

**Principal Investigators:** J. Jaime Miranda y Lorena Saavedra

**SIDISI No.:** 66215

**PI Version Date:** November 03, 2017 (Version 2.0)

*Please, introduce yourself orally before starting:*

*“Good morning/afternoon, my name is (say your name and show your credential). I am part of the staff of the Universidad Peruana Cayetano Heredia. We are carrying out Focus Groups to know about perceptions and knowledge regarding intake salt, and opinions and practices about the use of salt and, and also predisposition to reduce salt in food, and to collect ideas and suggestions as to how people might change their habits of salt consumption. If you decide to participate, we will ask you part of your time to be interviewed by our personnel. This session will take approximately one hour and will be held in a private location.*

*Before invite you, I would like to ask some questions about your general information and then start the session.”*

1. **Let’s talk about the foods**

- What did you eat for lunch yesterday?
  - Did you like it?
- In your house, who decide about what to prepare for eating?
  - (Investigate about who decides what to prepare for eating, it is not necessary who cooks. For example could be the mother who decide but the housewife is the one who cooks.)
- Who is in charge to cook in your house?
- Who buy the ingredients to cook?
- Usually, how many times do they go to buy ingredients to cook?
- Where do they buy it? (Investigate possible places that the group visit to buy the food)
- And why?
- Which of these groceries are always listed in your weekly buying?
- How many times in a week do you go out to eat with the members of your family?
  - Do you take the food prepared in the house to job, office or school?

1. **Let’s talk about the preparation of the foods:**

- Which are the secrets for having delicious foods? (Identify the most important element)
  - (Probe) apart from (the most frequent answer that gave you) are there something else to make the food tasty?
  - Who gave you that secret?
- If you had the opportunity to talk to someone that gives you tips to make your cooking best, to whom you would ask for help?
  - Among this 4 persons, who do you choose (male cooker, female cooker, old woman)
  - Why?
- For you, when you decide what to cook, what is the most important thing?
  - Flavor, money (price), time that the food takes to be cooked or healthy food?
  - Why?

1. **Let’s talk about the consumption of salt in your daily life**:

- When you cook…
  - How do you add salt to your foods? (Investigate if they use: salt shaker, tea spoon, little amount of salt, moderate quantity of salt)
  - And when you have your meals served, do you include salt?
- What do you know about the relationship between salt and health?
  - (If you don’t obtain answer) what could you hear about hypertension and the consumption of salt?
  - What are the good and the bad things of salt consumption?
- Have you ever tried to reduce the consumption of salt at home?
  - Why did you try? What did you do?
  - How did it go? What was the easiest thing and what was the hardest?
  - (If only you obtain few answers) for the ones who never tried, imagine that you want to do it. What do you do?
- Do you think there is a way to reduce the consumption of salt without changing the taste of the food?
  - (If it is a positive answer) how?
- How important is the salt to give flavor to the foods?
- There are some foods that have more and less salt.
  - Do you know what foods are salty?
- From these products, which are the most used in your house? (Artificial seasonings)
  - And from these others (spices)
- Once upon a time did you search information about healthy feeding?

**QUESTIONAIRE**

**CRONICAS - Center of Excellence in Chronic Diseases**

**Universidad Peruana Cayetano Heredia**

**APLICATION DATE: RECORD NUMBER:**

|  |  | **-** | **1** | **2** | **-** | **1** | **7** |
| --- | --- | --- | --- | --- | --- | --- | --- |

|  |  |  |  |  |  |
| --- | --- | --- | --- | --- | --- |

**Project: Scaling-up** [**and evaluating salt reduction policies and programs in Latin American countries**](http://en.cronicas-upch.pe/salt-reduction/)**.**

**Lima, November–2017**

**INDICATE BY CHECK MARK OR COMPLETE**

| **Section 1:**  **Sociodemographic Information** | | **Response** | | | **Check here** |
| --- | --- | --- | --- | --- | --- |
| 1 | Sex | 1 | Male | |  |
|  |  | 2 | Female | |  |
| 2 | Age in completed years  Years  7 Your age is: |  | **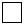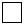** | Years | |
| 3 | What is your district of residence? |  | | | |
| 4 | What is your marital status? | 1 | Single | |  |
|  |  | 2 | Married | |  |
|  |  | 3 | Cohabiting with partner | |  |
|  |  | 4 | Separated | |  |
|  |  | 5 | Divorced | |  |
|  |  | 6 | Widow/widower | |  |
| 5 | What is the highest level of school you completed? | 1 | Primary | |  |
|  |  | 2 | Secondary | |  |
|  |  | 3 | Superior not universitary | |  |
|  |  | 4 | Superior universitary | |  |
| 6 | Are you currently working? | 1 | Yes | |  |
|  |  | 2 | No | |  |
| 7 | What is your current work activity? | 1 | Student | |  |
|  |  | 2 | Housework | |  |
|  |  | 3 | Self-employed | |  |
|  |  | 4 | Employee | |  |
|  |  | 5 | Others **🡪 Explain** | | |

**CHECK MARK A SINGLE ALTERNATIVE**

| **Section 2:**  **Foods and the salt in their family** | | **Response** | | **Check here** |
| --- | --- | --- | --- | --- |
| 8. | In your house, who decide about what to prepare for eating? | 1 | Mother |  |
|  |  | 2 | Father |  |
|  |  | 3 | Grandmother  Housekeeper |  |
|  |  | 4 | Housekeeper |  |
|  |  | 5 | Others **🡪 Explain** | |
| 9. | When you cook…  How do you add salt to your foods? | 1 | Fingers |  |
|  |  | 2 Cucharita (de té) | Teaspoon |  |
|  |  | 3 | Spoon |  |
|  |  | 4 | Soup ladle |  |
|  |  | 5 | Others **🡪 Explain** | |
| 10. | Usually, what do you use to give flavor to your food? | 1 | Use artificial seasonings (ajinomoto, cubitos, sibarita, etc.) |  |
|  |  | 2 | Add more salt |  |
|  |  | 3 | Use natural seasonings (pimiento, comino, kion, laurel, etc.) |  |
|  |  | 4 | Others **🡪 Explain** | |
| 11. | How frequently do you use artificial seasonings (Ajinomoto/Doña Gusta)? | 1 | Diary / interdiary |  |
|  |  | 2 | Twice a week |  |
|  |  | 3 | Rarely |  |
|  |  | 4 | I do not use |  |

**CHECK MARK A SINGLE ALTERNATIVE**

| **Section 3**  **Decisions we make when cooking** | | **Response** | | **Check here** |
| --- | --- | --- | --- | --- |
| 12. | What do you take into account when deciding what to cook? | 1 | The time |  |
|  |  | 2 | Flavor |  |
|  |  | 3 | Money |  |
|  |  | 4 | Healthy food |  |
| 13. | When you cook... Do you receive any suggestions from some members of their family? | 1 | Partner |  |
|  |  | 2 | Child |  |
|  |  | 3 | Grandmother / Grandfather |  |
|  |  | 4 | No body |  |
| 14. | Do you think there is a way to reduce the consumption of salt without changing the taste of the food? | 1 | Yes |  |
|  |  | 2 | No |  |
|  |  | 3 | Maybe |  |
|  |  | 4 | I eat less salt |  |

| **Section 4**  **Salt and health** | | **Response** | | **Check here** |
| --- | --- | --- | --- | --- |
| 15. | **CAN CHECK MARK VARIOUS ALTERNATIVES**  Do you know which diseases can be developed for the excessive consumption of salt? | 1 | Hypertension |  |
|  |  | 2 | Diabetes |  |
|  |  | 3 | Heart attacks |  |
|  |  | 4 | Cancer |  |
|  |  | 5 | I do not know |  |
|  |  | 6 | All mentioned |  |
|  |  | 7 | Others **🡪 Explain** | |
| 16. | If I tell you that your kids can develop diseases such as hypertension, diabetes or cancer for the consumption of salt, how do you feel? | 1 | Fear |  |
|  |  | 2 | Concern |  |
|  |  | 3 | Guilt |  |
|  |  | 4 | Others **🡪 Explain** | |

**CHECK MARK A SINGLE ALTERNATIVE**

| **Section 5**  **Some free ideas to know about salt and health** | | **Response** | | **Check here** | |
| --- | --- | --- | --- | --- | --- |
| 17. | Which of this channels/material would you prefer to learn something about healthy eating? | 1 | To participate in virtual cooking classes or in cooking sessions to learn how to cook with little amount of salt. | |  |
|  |  | 2 | Tutorial videos: YouTube or Facebook | |  |
|  |  | 3 | School wall newspaper | |  |
|  |  | 4 | A recipe book that inform you how to cook | |  |
|  |  | 5 | Chart/one magnet for the refrigerator that helps you to classify the food. | |  |
|  |  | 6 | Others **🡪 Explain** | | |

| 18. | How frequently do you use natural seasonings (albahaca, culantro, tomillo, perejil, laurel, etc.)? | 1 | Diary / interdiary | |  |
| --- | --- | --- | --- | --- | --- |
|  |  | 2 | Twice a week | |  |
|  |  | 3 | Rarely | |  |
|  |  | 4 | I do not use | |  |
| 19. | If you had the opportunity to talk to someone that gives you tips to make your cooking best, to whom you would ask for help? | 1 | Male cooker |  | |
|  |  | 2 | Female cooker |  | |
|  |  | 3 | Old woman |  | |
|  |  | 4 | Others **🡪 Explain** | | |
